# Systemic inflammatory profiles associated with early EEG abnormalities following ischemic stroke: a cohort study

**DOI:** 10.1007/s10072-026-09397-3

**Published:** 2026-09-16

**Authors:** Gabriele Prandin, Giovanni Furlanis, Edoardo Ricci, Laura Mancinelli, Emanuele Vincis, Magda Quagliotto, Michele Malesani, Gianpiero Farina, Paola Caruso, Giulia Mazzon, Marcello Naccarato, Marinella Tomaselli, Paolo Manganotti

**Affiliations:** Clinical Unit of Neurology, Department of Medicine, Surgery and Health Sciences, University of Trieste, Strada di fiume 447, Trieste, 34149 Italy

**Keywords:** Stroke, Inflammation, EEG, Slow activity, Epileptiform discharges

## Abstract

**Background:**

Post-stroke inflammation (PSI) contributes to acute brain injury and may influence early electroencephalographic (EEG) abnormalities after ischemic stroke. Although systemic inflammatory biomarkers have prognostic value, their association with specific EEG patterns - slow-wave activity (SA) and epileptiform discharges (ED) - remains unclear. We investigated the relationship between peripheral inflammatory markers and early EEG alterations following ischemic stroke.

**Methods:**

We conducted a single-center, retrospective observational study including 307 patients with acute supratentorial ischemic stroke who underwent EEG within 72 h from admission. Peripheral inflammatory biomarkers were measured 24 h after admission. EEGs were classified according to the presence of SA and/or ED. Multivariable logistic regression analyses were performed to identify independent predictors of SA and ED. Predictive performance was assessed using receiver operating characteristic (ROC) analysis.

**Results:**

Among patients without ED (*n* = 248), no inflammatory biomarker remained independently associated with SA after multivariable adjustment. Stroke severity and hemorrhagic transformation were the main independent predictors of SA. In the overall cohort, ED (*n* = 59) were independently associated with higher white blood cell count (OR 1.15, 95% CI 1.02–1.31), neutrophil count (OR 1.19, 95% CI 1.05–1.36), and NLR (OR 1.08, 95% CI 1.01–1.15), after adjustment for relevant clinical variables. Neutrophil count showed the highest discriminative ability for ED and significantly improved model performance when added to clinical predictors.

**Conclusions:**

Elevated neutrophil count is independently associated with early ED after ischemic stroke, supporting a role for systemic inflammation in acute post-stroke EEG hyperexcitability. In contrast, SA mainly reflects stroke severity and local complications.

**Supplementary Information:**

The online version contains supplementary material available at https://doi.org/10.1007/s10072-026-09397-3.

## Introduction

Post-stroke inflammation (PSI) plays a crucial role during the acute and subacute phases following a stroke [1, 2]. Among the various leukocyte subtypes, neutrophils and lymphocytes have been implicated in influencing clinical outcomes in distinct ways [3, 4]. Specifically, the neutrophil-to-lymphocyte ratio (NLR) and its associated indices have emerged as prognostic markers for mortality in various cardiovascular conditions and peripheral arterial occlusive disease [5]. Subsequent research has highlighted the prognostic significance of NLR and its derived indices in predicting mortality in patients with acute ischemic stroke [6]. Recent clinical studies have investigated the role of several inflammatory biomarkers in large vessel occlusion strokes. In particular, these biomarkers have been identified as powerful predictors of stroke outcome after mechanical thrombectomy (MT) [7], with sex-based differences [8], and as predictors of futile recanalisation in patients with successful MT [9], following distinct age-specific patterns. Finally, admission and 24-hour C-reactive protein (CRP) levels have been found to be associated with the development of atrial fibrillation detected after stroke [10].

In recent years, electroencephalography (EEG) has emerged as a valuable biomarker for predicting functional and clinical outcomes following stroke [11]. EEG is a non-invasive method with high temporal resolution, allowing for rapid assessment of real-time brain electrical activity and function [12]. Early EEG background asymmetry and the presence of interictal epileptiform activity are independent predictors of post-stroke epilepsy (PSE) development [13].

Moreover, slow activity and epileptic activity are correlated with worse stroke outcomes in a burden-dependent manner [15–18]. Therefore, a thorough understanding of the underlying pathophysiological mechanisms and the identification of potential biomarkers may enhance the ability to prevent epileptic activity, ultimately improving patient outcomes.

Oscillatory changes in acute stroke remain an important topic of discussion, as acute tissue damage, neuronal reactivity, and early network reorganization each play distinct roles in shaping immediate clinical symptoms and future prognosis. The objective of this study was to assess the impact of systemic inflammatory biomarkers on early EEG alterations following ischemic stroke. Specifically, we investigated the effect of PSI on the development of slow-wave activity (SA) and epileptiform discharges (ED) in EEG recordings obtained within 72 h of patient admission.

## Materials and methods

### Study design and patients

This was a single-center, observational, investigator-initiated, retrospective study that included patients with acute ischemic stroke admitted to the Stroke Unit of the University Hospital of Trieste (Italy) between January 2021 and April 2023. Ethical approval was obtained from the University Ethics Committee of the University of Trieste on 06/2025. Informed consent was obtained from participants or their legal representatives.

### Patient inclusion and exclusion criteria for the analysis

The inclusion criteria for patient selection were as follows: [1] ischemic stroke (with or without acute treatment); [2] age ≥ 18 years; [3] supratentorial stroke location; [4] EEG recording within 72 h of admission to the Stroke Unit (SU). The diagnosis of ischemic stroke was confirmed through both neuroradiological and clinical assessments, and the eligibility criteria for treatment were based on the ESO (European Stroke Organization) guidelines [18, 19]. Among the patients treated with reperfusion therapies, we included those who arrived at the hospital within 4.5 h of symptom onset or those with a favorable core/penumbra mismatch as determined by CT perfusion evaluation [20]. Specifically, we also included patients with an extended large vessel occlusion (LVO) window based on radiological findings (i.e., CT perfusion) or patients with unknown symptom onset time (wake-up strokes) who had a penumbra area greater than 50% of the total hypoperfused region [20].

For this analysis, we excluded stroke patients with hemorrhagic strokes (intracerebral hemorrhage or subarachnoid hemorrhage) and those with conditions that could potentially alter the EEG recordings, including previous strokes (ischemic or hemorrhagic), history of cognitive impairment, a history of epilepsy (with or without concomitant antiseizure medication), and patients who had experienced a seizure or status epilepticus prior to the EEG. Additionally, we excluded patients with conditions that might affect inflammatory biomarkers, for example the ongoing use of immunosuppressive drugs, current infections or infections that developed within 48 h of admission, chronic inflammatory conditions, active cancers, recent trauma, severe liver or kidney dysfunction (eGFR < 30 ml/min) as reported in the literature [7–11, 21, 22].

### Data collection

Demographic data, including age, sex, and comorbidities such as atrial fibrillation (AF), diabetes, arterial hypertension, hypercholesterolemia, and a history of coronary artery disease, were collected. Clinical features, including the NIHSS (National Institutes of Health Stroke Scale) score and mRS (modified Rankin Scale), were recorded at both admission and discharge. Additionally, the mRS and mortality rate at 3 months post-stroke were documented. An unfavorable functional outcome was defined as an mRS score between 3 and 6 at 90 days, while a favorable outcome was defined as an mRS score between 0 and 2 at 90 days. The mRS score at admission reflected the patient’s premorbid condition. Stroke etiology was assessed using the TOAST (Trial of Org 1072 in Acute Stroke Treatment) classification and categorized as large artery atherosclerosis (LAA), cardioembolic (CE), lacunar, other cause, or indeterminate/cryptogenic [23]. Stroke subtype was categorized following the Oxfordshire community stroke project (OCSP) as lacunar infarct (LACI), partial anterior circulation infarct (PACI), total anterior circulation infarct (TACI) or posterior circulation infarct (POCI) [24].

For all patients, the presence of hemorrhagic infarctions was evaluated using non-contrast computed tomography (NCCT) performed 24 h after admission to the Stroke Unit. Both asymptomatic and symptomatic hemorrhagic transformations, the latter defined by a clinical deterioration (acute worsening of NIHSS > 4 points) [25, 26], were recorded.

### EEG assessment

As part of standard clinical practice, our center routinely performs conventional EEG recordings (median duration: 30 min) in patients admitted to the Stroke Unit within the first 72 h following stroke onset. EEGs may be repeated in cases of clinical suspicion of seizures or to monitor severely abnormal tracings. Recordings are obtained digitally using 21 fixed Ag/AgCl electrodes placed according to the International 10–20 system, with the Be Plus PRO amplifier (EB Neuro, Florence, Italy). All recordings are performed in patients free of sedative medications [16]. 

Experienced neurophysiologists (GM, MT) subsequently review the EEGs and report any pathological findings based on the standardized international glossary [27], in particular the interictal EDs were identified according to the International Federation of Clinical Neurophysiology, (i.e. EDs were defined as di- or triphasic waves with sharp or spiky morphology distinct to background activity, typically followed by an associated slow after wave. Focal slowing was not considered as ED) [16]. Any uncertainties were resolved by consensus with an additional neurologist (GF).

EEG abnormalities, including slowing and interictal epileptiform activity, were classified as unilateral or bilateral and stratified based on their prevalence in the recording. Patients were included in the Slow Activity (SA) group only if they exhibited SA without any epileptiform discharges. Conversely, the Epileptiform discharges (ED) group comprised patients with epileptiform abnormalities, regardless of the presence or absence of SA.

### Blood samples

Venous blood samples were collected from all patients 24 h after admission. Inflammatory markers, including the white blood cell (WBC) count, neutrophil count (N), platelet count (P), lymphocyte count (L), and monocyte count (M), were analyzed in the local laboratory. Laboratory measurements were performed within 60 min of blood collection. The neutrophil-to-lymphocyte ratio (NLR) was calculated as the ratio of N to L [7–11].

### Statistical analysis

Two subgroup analyses were conducted to explore the role of inflammatory biomarkers. In the group of patients without ED, we compared those with SA to those without SA (normal EEG). Then, for the entire study population, we compared individuals with ED to those without ED. Detailed statistical tests used for each variable considered, as well as the criteria for inclusion in the multivariable analysis, are available in the Supplementary Materials. The SA analysis was restricted a priori to patients without ED in order to isolate the relationship between systemic inflammation and SA, avoiding confounding by the distinct pathophysiological process underlying epileptiform activity. Conversely, the ED analysis included both patients with and without concomitant SA, given that ED (irrespective of coexisting SA) represents the primary phenotype of clinical interest due to its established association with functional outcome. To assess the robustness of this analytic strategy, three sensitivity analyses were performed: (a) ED versus normal EEG only; (b) ED with SA versus isolated SA; and (c) SA (with or without ED) versus normal EEG only. In models (a) and (c), the covariate that was structurally invariant within one comparison group (SA and ED, respectively) was excluded due to quasi-complete separation.

The discriminative ability of inflammatory markers for the detection of ED was assessed by computing the area under the curve (AUC) through receiver operating characteristic (ROC) analysis. The predictive model included only variables that showed an independent association with ED in the multivariable analysis. No variables were pre-selected or forced into the model to enhance its performance.

## Results

**Fig. 1:**
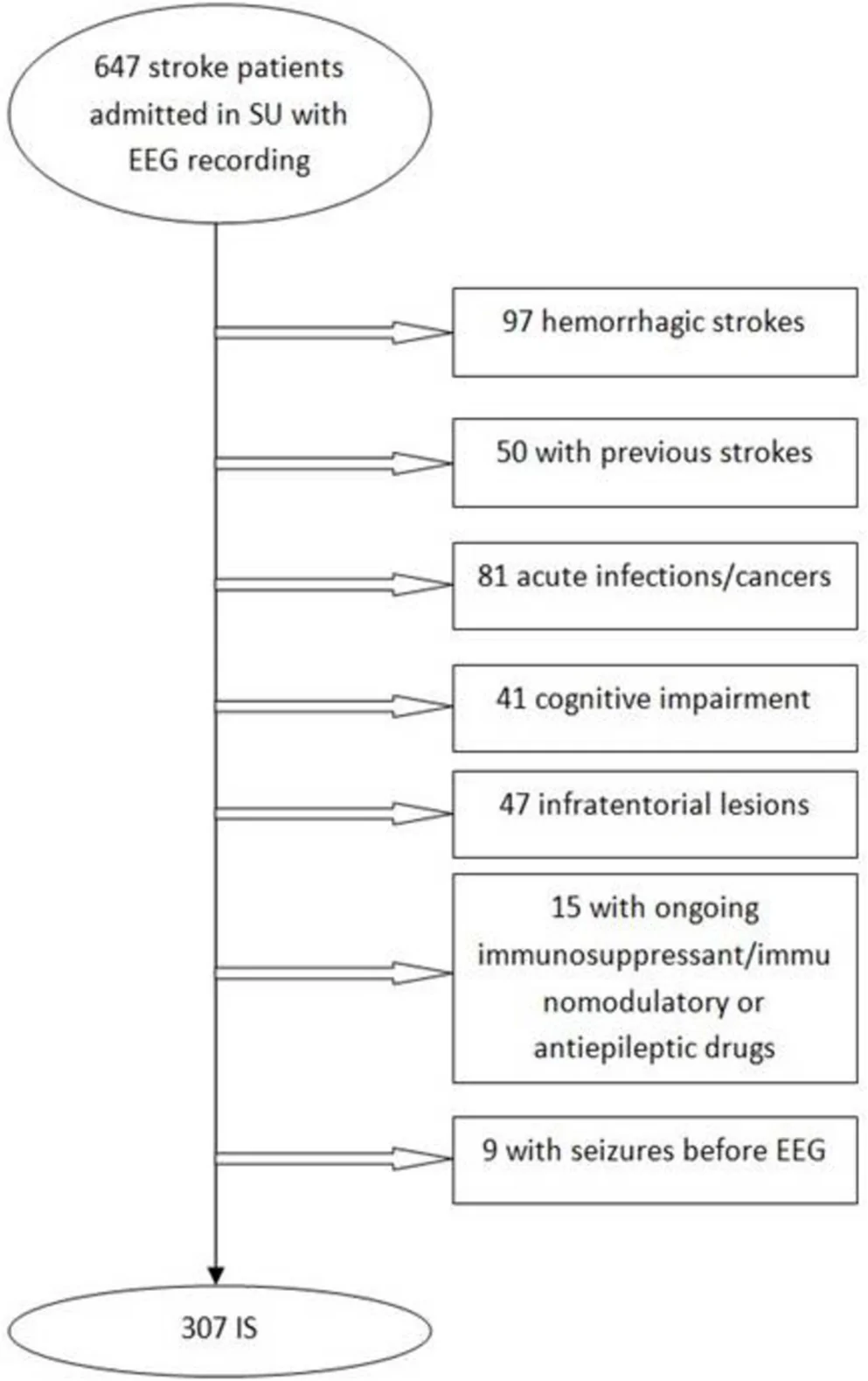
Study algorithm

After exclusion criteria, we evaluated data of 307 patients with ischemic stroke and EEG recording (Fig. 1) with a median (IQR) time from admission to EEG of 2 days (1–3 days).

In the cohort of patients without ED (*n* = 248), we compared clinical and radiological data between the patients cohort with or without SA (Table S1), in Table 1 are summarized the comparisons between the blood biomarkers investigated. Patients with SA have a higher prevalence of AF (33% vs. 20%), a more pronounced cardioembolic etiology (28% vs. 16%) and less lacunar etiology (18% vs. 37%), larger strokes as TACI (25% vs. 3%), a higher reperfusion treatment rate (64% vs. 43%), with more severe symptoms (median NIHSS 7 vs. 3) and a higher incidence of hemorrhagic transformation (HT) (19% vs. 4%). 90-day mortality and 90-day favorable outcome were significantly different in the two groups (8% vs. 1% and 68% vs. 92% respectively). About the EEG data, the admission-to-EEG timing was slightly different (*p* = 0.043).

Table 1 shows in SA cohort a significantly different inflammatory biomarkers levels, in particular the Neutrophil count (median 5.49 vs. 4.56, *p* = 0.003), Lymphocyte count (median 1.64 vs. 1.84, *p* = 0.006), Eosinophil count (median 0.09 vs. 0.13, *p* = 0.007) and the NLR (median 3.30 vs. 2.50, *p* < 0.001).

**Table 1:** Blood biomarkers between SA and no-SA cohort

	No-SA (*N* = 108)	SA (*N* = 140)	*p*
24 h blood test [median (IQR)]			
WBC	7.61 (6.42–9.22)	8.35 (6.56–9.98)	0.052
Neutrophils	4.56 (3.75–6.02)	5.49 (4.12–7.44)	**0.003**
Lymphocytes	1.84 (1.41–2.30)	1.64 (1.10–1.98)	**0.006**
Monocytes	0.71 (0.56–0.80)	0.71 (0.55–0.87)	0.556
Eosinophils	0.13 (0.07–0.23)	0.09 (0.03–0.16)	**0.007**
CRP	4.2 (1.8-9.0)	4.6 (2.3–10.4)	0.293
ESR	20 (12–32)	19 (11–32)	0.941
NLR	2.50 (1.80–3.68)	3.30 (2.26–5.58)	**< 0.001**

The variables resulted significantly different in the groups comparisons were included in a univariate analysis with SA (Table S2). Variables significantly associated in the univariate regression were included in the multivariable model, in order to adjust the possible confounders for each inflammatory biomarker (Table 2). In the model with NLR, remains significantly associated with the outcome only admission NIHSS (OR 1.177, CI95% 1.086–1.276, *p* < 0.001) and HT (OR 4.247, CI95% 1.228–14.690, *p* = 0.022); in the model including neutrophils, remains significantly associated admission NIHSS (OR 1.178, CI95% 1.086–1.278, *p* < 0.001) and HT (OR 4.095, CI95% 1.205–13.915, *p* = 0.024); in the model including lymphocytes remains significantly associated admission NIHSS (OR 1.164, CI95% 1.077–1.259, *p* < 0.001) and HT (OR 4.013, CI95% 1.184–13.607, *p* = 0.026). No significant association was found between inflammatory biomarkers and SA.

**Table 2:** Multivariable analysis to predict SA (binary logistic regression)

	SA*		
	OR	CI 95%	*p*
NLR	0.970	0.878–1.072	0.554
Neutrophils	0.955	0.816–1.118	0.568
Lymphocytes	0.884	0.572–13.660	0.578

*Adjusted for atrial fibrillation, hemorrhagic transformation, admission NIHSS, acute treatment, OCSP, TOAST

In the second part of our analysis, we evaluated the whole sample (*n* = 307) including also patients with ED in the EEG records. In Table S3 and Table 3 are summarized the clinic-radiological characteristics and blood biomarkers in patients with or without ED on the acute EEG trace.

**Table 3:** Blood biomarkers between ED and no-ED cohort

	No ED (*N* = 248)	ED (*N* = 59)	*p*
24h blood test (median [IQR])			
WBC	8.06 (6.49–9.62)	8.93 (7.37–12.04)	0.001
Neutrophils	5.17 (3.88–6.96)	6.57 (4.69–9.83)	**< 0.001**
Lymphocytes	1.69 (1.21–2.17)	1.51 (0.95–2.19)	0.159
Monocytes	0.71 (0.55–0.87)	0.70 (0.50–0.83)	0.520
Eosinophils	0.10 (0.04–0.19)	0.04 (0.01–0.13)	**0.001**
CRP	4.5 (2.1–9.8)	6.7 (3.5–10.4)	**0.031**
ESR	20 (11–32)	20 (11–32)	0.905
NLR	2.92 (2.07-5.00)	3.89 (2.41–7.30)	**0.005**

As inflammatory biomarkers resulted significantly different between ED and no-ED cohorts, we performed a multivariable regression analysis to assess their association with the prespecified endpoint, adjusting for confounders (sex, atrial fibrillation, OCSP subtype, admission NIHSS, and SA) for each inflammatory biomarker separately, as reported in Table S4 and Table 4. After adjustment, neutrophils, WBC, and NLR remained independently associated with ED development.

**Table 4:** Multivariable analysis to predict ED (binary logistic regression)

	ED*		
	OR	CI 95%	*p*
WBC	1.154	1.020–1.306	**0.021**
Neutrophils	1.189	1.045–1.355	**0.009**
Eosinophils	0.305	0.022–4.234	0.377
NLR	1.076	1.011–1.145	**0.021**

*Adjusted for sex, atrial fibrillation, OCSP subtype, admission NIHSS, SA

In order to select the best inflammatory biomarker for ED prediction, we performed a ROC analysis, which showed that neutrophils had a higher area under the curve (AUC) compared to WBC and NLR (Table S5).

Figure S2 and Table S6 illustrate the superior performance of the combined model (significant clinical variable (CV) plus neutrophils) compared to CV alone, following multivariable analysis (sex + slow activity). The AUCs of the two curves differ significantly according to the DeLong test (*p* = 0.040).

**Fig. 2:**
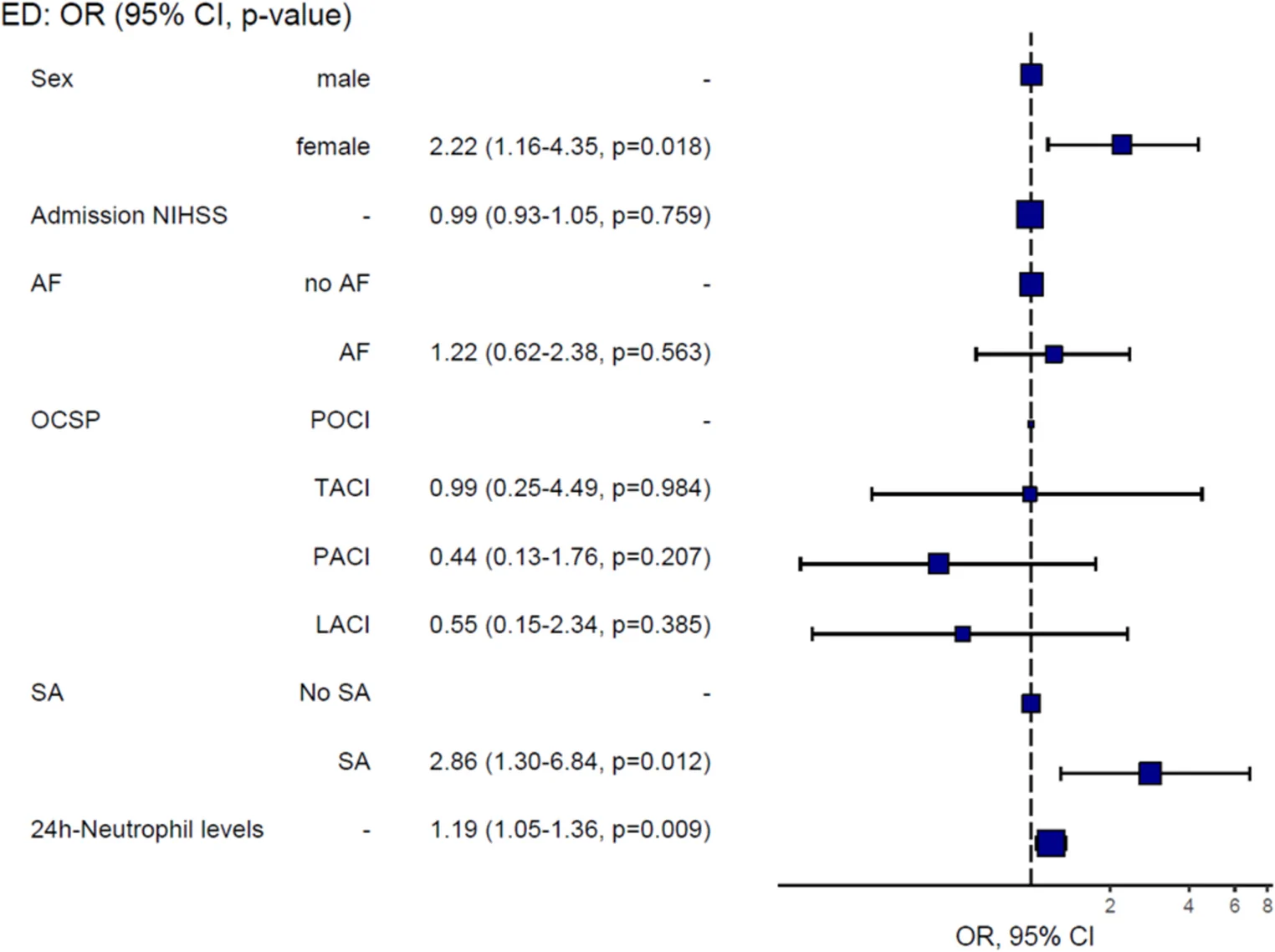
Forest plot for multivariable analysis to predict ED with 24 h-Neutrophil count

Figure 2 summarizes the multivariable analysis of ED development in relation to neutrophil levels. Along with neutrophil count, female sex and the presence of SA remained associated with ED.

**Fig. 3:**
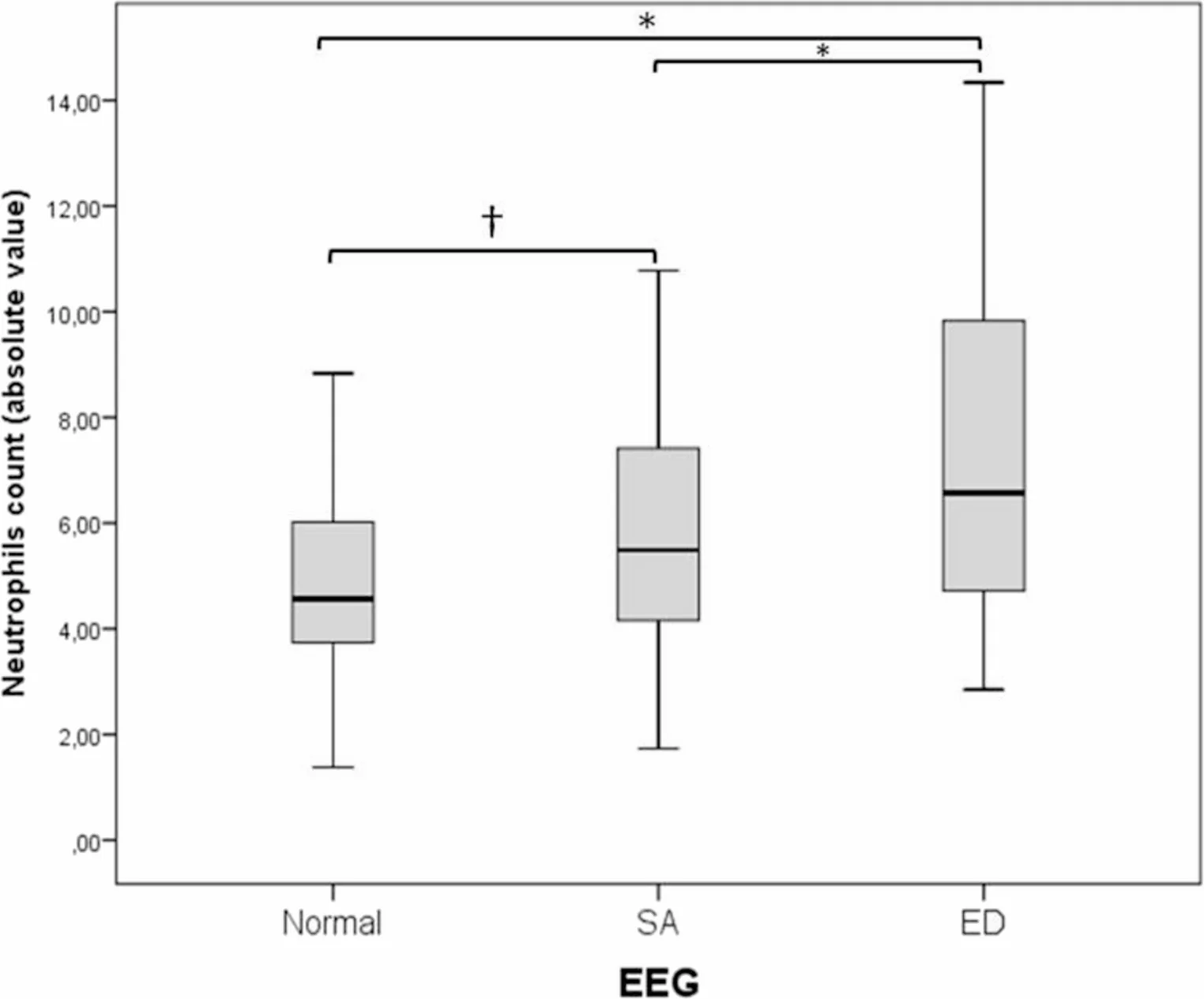
Neutrophil count in different EEG traces after ischemic stroke. Legend: Absolute value for Neutrophils count is expressed as *10^3; SA= slow activity, ED: epileptic discharges with or without accompanying SA, *p < 0.001, †*p* < 0.05

Figure 3 illustrates neutrophil count in relation to the various EEG characteristics analyzed: normal EEG, SA, and ED (with or without accompanying SA).

Three sensitivity analyses were performed to assess the robustness of the primary analytic strategy (Table S7). Neutrophil count remained independently associated with ED when the comparator was restricted to normal EEG only (OR 1.199, 95% CI 1.016–1.415, *p* = 0.032) and when the comparison was limited to ED + SA versus isolated SA (OR 1.204, 95% CI 1.052–1.377, *p* = 0.007). In contrast, neutrophil count remained unassociated with SA even when the SA group was broadened to include patients with concomitant ED (OR 1.075, 95% CI 0.943–1.225, *p* = 0.279).

## Discussion

This study aimed to investigate the possible role of systemic inflammatory biomarkers in the acute EEG alteration in a highly selected population. We found that SA may not be influenced by post-stroke cellular inflammation, whereas ED was independently associated with 24 h-Neutrophil levels. The presence of ED in acute EEG has been recently linked to poor 90-day functional outcome [16], and clarifying the biological processes underlying this association may help identify strategies to improve medium- and long-term outcomes.

Several pathways have been proposed to explain the association between systemic inflammation and epileptic activity [28]. It should be noted, however, that our inflammatory assessment relied exclusively on routine hematological biomarkers, which offer only an indirect estimate of the systemic inflammatory response. Following a brain insult, the production of various interleukins (such as IL-1, IL-6, and TNF-α) leads to the breakdown of the blood-brain barrier (BBB), accompanied by activation of the endothelium and microglia. Leukocyte-endothelium interactions at the BBB facilitate vascular leakage and infiltration of inflammatory cells into the brain parenchyma, predominantly neutrophils. These cells secrete matrix metalloproteinase-9 (MMP-9), which further promotes leukocyte infiltration and perpetuates the inflammatory response. Moreover, it has been described in epileptic patients the possible role of perfusional characteristics [29]; in that study, the authors found a higher fMRI signal concordant with the presumed seizure onset zone that appeared roughly 26 s before the full-blown clinical seizure. Local inflammation exerts immediate non-transcriptional effects on ion channels, neurotransmitter release, and glutamate receptors, thereby increasing neuronal excitability. These processes have been proposed to contribute to the development of epilepsy.

Our finding that neutrophils were independently associated with ED detected on early post-stroke EEG is compatible with this proposed model of epileptogenesis (although our cross-sectional design cannot establish whether neutrophils play a mechanistic role or instead represent a marker of injury severity, cortical damage, or blood-brain barrier disruption, all of which may independently predispose to epileptiform activity). Neutrophil counts may also serve as a nonspecific marker of stroke severity or extent of cortical involvement, both of which are independently linked to epileptiform activity; our analysis cannot fully disentangle these possibilities from a direct pro-epileptogenic effect of neutrophils. No other inflammatory biomarkers were found to be associated with ED. It is interesting instead that we did not find an association between serum inflammatory biomarkers and SA. A possible role of other inflammatory mediators such as IL-1β and TNF in enhancing SA has been proposed [9], but it is possible that their effect is mediated by local mechanisms (e.g., edema development) rather than by recruitment of white blood cells from the bloodstream. Previous clinical studies with rigorous inclusion/exclusion criteria [7–11], have found a significant association between ischemic stroke outcome and inflammatory biomarkers, particularly neutrophil and lymphocyte counts (most commonly expressed as the NLR) measured at 24 h after admission [7–10]. A possible explanation for the distinct biomarker identified in the present study is that neutrophil activation, rather than the broader lymphocyte-mediated component captured by the NLR, may specifically drive this pathway. Taken together, these findings suggest that post-stroke inflammation does not act through a single pathway, but exerts heterogeneous effects depending on the outcome considered.

Although neutrophil count showed the best discriminative performance among the inflammatory biomarkers evaluated (AUC = 0.654, 95% CI 0.573–0.736, *p* < 0.001), its discriminative ability in isolation remains modest, and this finding should be interpreted with caution. Notably, the addition of neutrophil count to a model based on clinical variables alone (sex and slow-wave activity, AUC = 0.701, 95% CI 0.633–0.768) improved discrimination to an AUC of 0.748 (95% CI 0.673–0.819), suggesting that its clinical value may lie primarily in complementing, rather than replacing, clinical predictors within a multivariable prognostic model. External validation in independent cohorts will be needed before any clinical application, including incorporation into decision-support tools, can be considered.

In the sensitivity analysis, Neutrophil count remained independently associated with ED across all alternative definitions, including when the comparator was restricted to normal EEG only and when the comparison was limited to patients with concomitant SA, with effect sizes closely matching the primary analysis. In contrast, neutrophil count remained unassociated with SA even when the SA group was broadened to include patients with concomitant ED. These findings support a specific association between neutrophil-driven inflammation and epileptiform activity, rather than a generic relationship between systemic inflammation and EEG abnormalities more broadly, and indicate that our primary findings are not an artifact of the asymmetric group definitions.

In addition to neutrophil levels, we found a significant association between female sex and the presence of EDs. This finding contrasts with a previous meta-analysis [30], which reported a mild protective effect of female sex against the development of post-stroke seizures in patients with large vessel occlusions treated with mechanical thrombectomy. However, previous studies conducted at our center revealed a higher proportion of female patients within cohorts exhibiting post-stroke epileptiform activity [16, 17]. Despite differences in the study populations, further research is needed to clarify the role of sex in the development of both seizures and acute EEG epileptiform abnormalities.

SA was not found associated with post stroke inflammation even if significantly higher levels of different biomarkers were found in the comparison with normal EEG. A pivotal role seems to be played by the stroke severity (admission NIHSS) and local complication (hemorrhagic transformation). Both of them demonstrate a higher stroke extension and a worsening of the damage which is in line with previous research [15, 31].

This study has several limitations. First, it is a retrospective, cross-sectional analysis, which does not allow causal inference and may be influenced by unmeasured confounding variables; associations between inflammatory biomarkers and EEG findings should therefore be interpreted as hypothesis-generating rather than mechanistic. Second, the study was conducted at a single center, which may limit the generalizability of our findings. Generalizability is further limited by the strict inclusion and exclusion criteria, which do not fully reflect the typical stroke-unit population encountered in daily clinical practice. In particular, the exclusion of patients with previous stroke, infections, chronic inflammatory disorders, cancer, epilepsy, and other common comorbidities, although necessary to improve internal validity and reduce confounding related to inflammatory status, means that our findings may not be directly applicable to the broader, more heterogeneous population typically admitted to stroke units. Third, EEG features were evaluated through visual inspection by experienced neurophysiologists, introducing the possibility of inter-rater variability. Formal inter-rater reliability measures (e.g., Cohen’s kappa) were not calculated in the present study, and this should be acknowledged as a limitation. Future studies incorporating structured agreement analysis between EEG readers would help to further strengthen the methodological rigor of this line of research. Fourth, the prevalence of epileptiform abnormalities in the pre-stroke population remains unknown, and the use of point-of-care EEG rather than continuous monitoring may have reduced the sensitivity for detecting transient epileptiform patterns. Moreover, data on distinct epileptiform patterns were not collected, thereby limiting our ability to distinguish the differential effects of neutrophils across these patterns. Additionally, our inflammatory assessment was limited to routine hematological biomarkers (e.g., neutrophil counts), which are clinically accessible but provide only an indirect readout of the underlying inflammatory response; cytokines, markers of blood-brain barrier integrity, and direct indices of microglial activation were not measured, and the mechanistic framework discussed above should therefore be interpreted as a plausible but unverified explanatory model.

Despite these limitations, the study contributes valuable insights into the role of inflammatory biomarkers in a homogenous patient population selected using stringent inclusion criteria, both from an EEG and inflammation standpoint. We hope that this work will stimulate further interest in the field and serve as a foundation for evaluating the impact of future anti-inflammatory treatments.

## Conclusions

Elevated neutrophil count is independently associated with early epileptiform discharges after ischemic stroke, supporting a role for systemic inflammation in acute post-stroke epileptogenesis. In contrast, SA appears to be driven primarily by stroke severity and local complications rather than systemic inflammatory burden.

## Supplementary Information

Below is the link to the electronic supplementary material.

Supplementary Material 1

Supplementary Material 2

Supplementary Material 3

## Data Availability

The data will only be made available from the corresponding author upon reasonable request.
