# Supplementary material for: Systemic inflammatory profiles associated with early EEG abnormalities following ischemic stroke: a cohort study
Source: Neurol Sci. 2026 Sep 16;47(10):788. doi: 10.1007/s10072-026-09397-3 (PMC13578100; doi:10.1007/s10072-026-09397-3)
Supplement: Supplementary file 1 [file 10072_2026_9397_MOESM1_ESM.docx]

**Figure s1:** Receiver operating characteristics (ROC) curve for inflammatory biomarkers for predicting ED

**Figure S2:** Receiver operating characteristics (ROC) curve for predicting ED with Clinical Variables (CV) alone and including inflammatory biomarker (neutrophils).
