# Supplementary material for: Systemic inflammatory profiles associated with early EEG abnormalities following ischemic stroke: a cohort study
Source: Neurol Sci. 2026 Sep 16;47(10):788. doi: 10.1007/s10072-026-09397-3 (PMC13578100; doi:10.1007/s10072-026-09397-3)
Supplement: Supplementary file 2 [file 10072_2026_9397_MOESM2_ESM.docx]

**Table S1**: Clinical and radiological characteristics in patients with and without SA.

|  | **No SA**  **(N=108)** | **SA**  **(N=140)** | **p** |
| --- | --- | --- | --- |
| **Demographics** |  |  |  |
| Age, years [median (IQR)] | 74 (63-81) | 74 (64-84) | 0.265 |
| Female sex [n, (%)] | 41 (38) | 64 (46) | 0.221 |
| Hypertension [n, (%)] | 78 (72) | 98 (70) | 0.702 |
| Diabetes mellitus [n, (%)] | 24 (22) | 32 (23) | 0.906 |
| Hypercholesterolemia [n,(%)] | 64 (59) | 84 (60) | 0.906 |
| Atrial fibrillation [n, (%)] | 21 (20) | 46 (33) | **0.018** |
| Chronic Kidney disease [n, (%)] | 10 (9) | 19 (13) | 0.295 |
| Coronary artery disease [n, (%)] | 13 (12) | 21 (15) | 0.501 |
| Congestive heart failure [n, (%)] | 8 (7) | 17 (12) | 0.219 |
| Current smoking [n, (%)] | 26 (24) | 24 (17) | 0.177 |
| TOAST etiology [n, (%)]  CE  LAA  Lacunar  Other  Cryptogenic/indeterminate | 17 (16)  15 (14)  40 (37)  6 (6)  30 (28) | 39 (28)  15 (11)  25 (18)  8 (6)  53 (38) | **0.005** |
| OCSP classification [n, (%)]  TACI  PACI  LACI  POCI | 3 (3)  58 (53)  40 (37)  7 (7) | 35 (25)  73 (52)  25 (18)  7 (5) | **<0.001** |
| **Stroke characteristics and outcome** |  |  |  |
| Admission NIHSS [median (IQR)] | 3 (1-5) | 7 (3-12) | **<0.001** |
| Acute Treatment [n, (%)] | 46 (43) | 89 (64) | **0.001** |
| Hemorrhagic transformation [n, (%)] | 4 (4) | 26 (19) | **<0.001** |
| 90-day mRS 0-2 [n, (%)] | 99 (92) | 95 (68) | **<0.001** |
| 90-day mortality [n, (%)] | 1 (1) | 11 (8) | **0.014** |
| **EEG characteristics** |  |  |  |
| Admission to first EEG (days) [median (IQR)] | 2 (1-4) | 2 (1-3) | **0.043** |
| Prevalence of slow activity [n, (%)]  90% continuous  50–89% abundant  10–49% frequent  1–9% occasional  <1% rare |  | 2 (1)  11 (8)  33 (24)  38 (27)  56 (40) |  |
| Slow activity asymmetry [n, (%)] |  | 90 (64) |  |

**Table S2:** univariate association of variables to EEG with SA (binary logistic regression)

|  | **UNIVARIATE** | |
| --- | --- | --- |
|  | **OR (95% CI)** | **p** |
| **Acute Treatment** | 2.352 (1.380-3.841) | **0.001** |
| **AF** | 2.027 (1.121-3.668) | **0.019** |
| **OCSP**  TACI  PACI  LACI  POCI | 11.667 (2.410-56.487)  1.259 (0.418-3.792)  0.625 (0.196-1.995)  Ref | **<0.001**  **0.002**  0.683  0.427 |
| **TOAST**  CE  LAA  Lacunar  Other  Cryptogenic/indeterminate | 1.299 (0.629-2.680)  0.566 (0.243-1.317)  0.354 (0.181-0.692)  0.755 (0.239-2.382)  Ref | **0.006**  0.480  0.186  **0.002**  0.631 |
| **HT** | 5.930 (2.002-17.561) | **0.001** |
| **NIHSS on admission** | 1.206 (1.127-1.290) | **<0.001** |
| **NLR** | 1.143 (1.042-1.254) | **0.005** |
| **Neutrophils** | 1.206 (1.067-1.363) | **0.003** |
| **Lymphocytes** | 0.640 (0.448-0.915) | **0.014** |
| **Eosinophils** | 0.499 (0.100-2.530) | 0.398 |
| **Admission to EEG time** | 1.011 (0.952-1.074) | 0.726 |

**Table S3**: Clinical and radiological characteristics in patients with and without ED.

|  | **No ED**  **(N=248)** | **ED**  **(N=59)** | **p** |
| --- | --- | --- | --- |
| **Demographics** |  |  |  |
| Age, years [median (IQR)] | 74 (63-82) | 79 (65-87) | 0.051 |
| Female sex [n, (%)] | 105 (42) | 39 (66) | **0.001** |
| Hypertension [n, (%)] | 176 (71) | 45 (76) | 0.415 |
| Diabetes mellitus [n, (%)] | 56 (23) | 14 (24) | 0.850 |
| Hypercholesterolemia [n,(%)] | 148 (60) | 33 (56) | 0.599 |
| Atrial fibrillation [n, (%)] | 67 (27) | 26 (44) | **0.010** |
| Chronic Kidney disease [n, (%)] | 29 (12) | 8 (14) | 0.692 |
| Coronary artery disease [n, (%)] | 34 (14) | 9 (15) | 0.759 |
| Congestive heart failure [n, (%)] | 25 (10) | 6 (10) | 0.984 |
| Current smoking [n, (%)] | 50 (20) | 7 (12) | 0.141 |
| TOAST etiology [n, (%)]  CE  LAA  Lacunar  Other  Cryptogenic/indeterminate | 56 (23)  30 (12)  65 (26)  14 (6)  83 (34) | 21 (36)  5 (9)  10 (17)  3 (5)  20 (34) | 0.242 |
| OCSPclassification [n, (%)]  TACI  PACI  LACI  POCI | 38 (15)  131 (53)  65 (26)  14 (6) | 24 (41)  21 (36)  10 (17)  4 (7) | **<0.001** |
| **Stroke characteristics and outcome** |  |  |  |
| Admission NIHSS [median (IQR)] | 4 (2-9) | 9 (4-18) | **0.001** |
| Treated patients [n, (%)] | 135 (54) | 40 (67) | 0.062 |
| Hemorrhagic transformation [n, (%)] | 30 (12) | 9 (15) | 0.513 |
| 90 days mRS 0-2 [n, (%)] | 194 (78) | 26 (44) | **<0.001** |
| 90 day mortality [n, (%)] | 12 (5) | 6 (10) | 0.127 |
| **EEG characteristics** |  |  |  |
| Admission to first EEG (days) [median (IQR)] | 2 (1-4) | 2 (1-2) | 0.074 |
| Prevalence of epileptiformactivity [n, (%)]  90% continuous  50–89% abundant  10–49% frequent  1–9% occasional  <1% rare |  | 6 (10)  0 (0)  16 (27)  11 (18)  26 (44) |  |
| Epileptiform discharges asymmetry [n, (%)] |  | 43 (73) |  |
| Presence of slow wave activity [n, (%)] | 140 (57) | 50 (85) | **<0.001** |
| Prevalence of slow activity [n, (%)]  90% continuous  50–89% abundant  10–49% frequent  1–9% occasional  <1% rare | 2 (1)  11 (8)  33 (24)  38 (27)  56 (40) | 4 (8)  8 (16)  22 (44)  10 (20)  6 (12) | **<0.001** |
| Slow activity asymmetry [n, (%)] | 90 (64) | 44 (88) | **0.002** |

**Table S4:** univariate association of variables to EEG with ED (binary logistic regression)

|  | **UNIVARIATE** | |
| --- | --- | --- |
|  | **OR (95% CI)** | **p** |
| **Female Sex** | 2.656 (1.465-4.815) | **0.001** |
| **AF** | 2.128 (1.185-3.822) | **0.011** |
| **OCSP**  TACI  PACI  LACI  POCI | 2.211 (0.651-7.511)  0.561 (0.168-1.868)  0.538 (0.147-1.967)  Ref | **<0.001**  0.204  0.346  0.349 |
| **Slow wave presence** | 4.286 (2.019-9.098) | **<0.001** |
| **NIHSS on admission** | 1.088 (1.046-1.131) | **<0.001** |
| **WBC** | 1.228 (1.103-1.368) | **<0.001** |
| **Neutrophils** | 1.266 (1.138-1.409) | **<0.001** |
| **Eosinophils** | 0.045 (0.003-0.729) | **0.029** |
| **NLR** | 1.111 (1.050-1.176) | **<0.001** |
| **CRP** | 1.018 (0.997-1.039) | 0.096 |

**Table S5:** Receiver operating characteristics (ROC) analysis for inflammatory biomarkers for predicting ED

|  | **Area** | **CI 95%** | **p value** |
| --- | --- | --- | --- |
| WBC | 0.641 | 0.561-0.720 | **0.001** |
| Neutrophils | 0.654 | 0.573-0.736 | **<0.001** |
| NLR | 0.618 | 0.532-0.704 | **0.005** |

**Table S6**: Receiver operating characteristics (ROC) analysis for predicting ED

|  | **Area** | **CI 95%** | **p** |
| --- | --- | --- | --- |
| CV | 0.701 | 0.633-0.768 | <0.001 |
| CV+Neutrophils | 0.748 | 0.673-0.819 | <0.001 |

Legend: CV= clinical variables (sex+SA)

**Table S7.** Sensitivity analyses for the association between inflammatory biomarkers and EEG abnormalities under alternative group definitions

| **Analysis** | **Comparison** | **N (cases vs. controls)** | **Biomarker** | **Univariate p (Mann-Whitney)ᵃ** | **Adjusted OR (95% CI)ᵇ** | **Adjusted p** |
| --- | --- | --- | --- | --- | --- | --- |
| (a) | ED vs. normal EEG only | 59 vs. 108 | Neutrophils | <0.001 | 1.199 (1.016–1.415) | 0.032 |
| (b) | ED+SA vs. isolated SA | 50 vs. 140 | Neutrophils | 0.001 | 1.204 (1.052–1.377) | 0.007 |
| (c) | SA (±ED) vs. normal EEG only | 190 vs. 108 | Neutrophils | <0.001 | 1.075 (0.943–1.225) | 0.279 |

ᵃ Mann-Whitney U test comparing biomarker distributions between groups.
ᵇ Binary logistic regression adjusted for sex, atrial fibrillation, OCSP subtype, and admission NIHSS. SA and ED were not included as covariates in analyses (a) and (c) respectively, due to quasi-complete separation.
