# Supplementary material for: Systemic inflammatory profiles associated with early EEG abnormalities following ischemic stroke: a cohort study
Source: Neurol Sci. 2026 Sep 16;47(10):788. doi: 10.1007/s10072-026-09397-3 (PMC13578100; doi:10.1007/s10072-026-09397-3)
Supplement: Supplementary file 3 [file 10072_2026_9397_MOESM3_ESM.docx]

***Supplementary statistical analysis***

Statistical analyses were conducted using SPSS statistical software (version 22, IBM Inc.), Jamovi ver 2.6.44. and R Studio (version 2024.4). The Kolmogorov-Smirnov test was applied to assess the normality of the variable distributions. Continuous variables with a normal distribution are presented as means and standard deviations (SD), while those with a skewed distribution are reported as medians and interquartile ranges (IQR), defined by the 1st and 3rd quartiles. Categorical variables are expressed as counts and percentages (%).

Differences between groups were assessed using the Mann-Whitney U-test for skewed variables, Pearson's chi-square test for categorical variables, or Fisher's Exact Test as appropriate. A p-value of < 0.05 was considered statistically significant. Variables that showed significant differences between groups were further analyzed using univariate and multivariate logistic regression to control for potential confounding factors.
